# CLC3 regulates V-ATPase to enhance lysosomal degradation and cisplatin resistance in cervical cancer cells

**DOI:** 10.1038/s41420-025-02876-0

**Published:** 2025-12-03

**Authors:** Chuyun Chen, Fubin Zhang, Jiayi Shen, Qi Zheng, Zhiyun Zhang, Shun Lu, Lixiao Liu, Tianhong Zhu, Yongming Du, Yutao Guan

**Affiliations:** 1 Center for Reproductive Medicine, the First Affiliated Hospital of Ningbo University, Ningbo, Zhejiang Province China; 2 Department of Obstetrics and Gynecology, the First Affiliated Hospital of Ningbo University, Ningbo, Zhejiang Province China; 3 Health Science Center, Ningbo University, Ningbo, Zhejiang Province China

**Keywords:** Cancer, Genetics

## Abstract

Chemoresistance remains a major challenge in cervical cancer (CVC) treatment. Lysosomal function, mediated by V-ATPase, is critical in cancer progression and drug resistance. CLC3, a chloride channel that regulates lysosomal acidification, may contribute to chemoresistance by modulating V-ATPase activity. This study aims to investigate the role of CLC3 in modulating lysosomal function, chemoresistance, and tumorigenesis in CVC. CLC3 expression in CVC cell lines was assessed, and chemoresistance was evaluated using IC50 calculations for cisplatin, paclitaxel, and 5-FU. Effects of CLC3 downregulation or overexpression on lysosomal pH, autophagy, apoptosis, cell proliferation, cell cycle progression, and tumor stemness were analyzed. A general V-ATPase inhibitor was used to assess changes in lysosomal pH and protein degradation, while a2v-mAb was applied to investigate the interaction between CLC3 and specific V-ATPase subunits. In vivo, a mouse xenograft model was used to assess the effects of CLC3 modulation on tumor growth and response to chemoresistance. CLC3 was upregulated in CVC cells, reducing chemosensitivity. Overexpression of CLC3 enhanced cytosolic alkalinization, lysosomal acidification, and protein degradation while inhibiting autophagy and apoptosis independently. CLC3 promoted cell proliferation and tumor stemness via V-ATPase activity, particularly ATP6V1A. CLC3 knockdown combined with V-ATPase inhibition decreased proliferation and increased cisplatin sensitivity. In vivo, CLC3 knockdown with cisplatin reduced tumor volume and increased apoptosis, whereas overexpression promoted cisplatin resistance. CLC3 plays a pivotal role in chemoresistance and tumor progression in CVC by regulating lysosomal function via V-ATPase. Targeting CLC3 and its downstream pathways may provide novel therapeutic strategies to overcome chemoresistance.

## Introduction

Cervical cancer (CVC) is the fourth most common cancer among women worldwide, representing a substantial portion of global female cancer cases. Despite significant advances in prevention through human papillomavirus (HPV) vaccination and early detection via screening programs, CVC remains a major public health concern, particularly in low- and middle-income regions where access to these interventions is limited [1]. Managing advanced CVC is particularly challenging due to the limited efficacy of current therapeutic options and the frequent emergence of chemoresistance, a significant hurdle in the treatment of advanced-stage disease.

Chemoresistance in CVC is a complex, multifactorial phenomenon involving genetic, epigenetic, and microenvironmental factors. One emerging area of interest in cancer research is the role of lysosomes in drug resistance. Enhanced lysosomal acidification supports cancer cell survival in acidic microenvironments and contributes to the development of resistance to chemotherapy [2]. Traditionally recognized as the cell’s degradative organelles, lysosomes are now understood to play critical roles in various cellular processes beyond waste disposal, including nutrient sensing, metabolic regulation, and cell death [3]. Central to lysosomal function is the vacuolar-type H + -ATPase (V-ATPase), a multi-subunit enzyme complex that pumps protons into the lysosome, maintaining the acidic environment necessary for the optimal activity of lysosomal enzymes. This acidic environment is crucial for activating hydrolases and facilitating the degradation of macromolecules [4, 5]. The V-ATPase complex comprises two main domains: the V1 domain, responsible for ATP hydrolysis, and the V0 domain, which translocates protons across the lysosomal membrane [6]. Disruption of V-ATPase function and lysosomal pH imbalance have been associated with aging and neurodegenerative diseases, such as Parkinson’s and Alzheimer’s diseases [7].

V-ATPase activity is crucial for maintaining lysosomal acidification and plays a key role in various cellular processes that promote cancer cell survival, including nutrient sensing, signal transduction, and cellular metabolism [8–10]. In cancer progressions, the acidification of lysosomes is often upregulated due to increased V-ATPase activity, which lowers lysosomal pH and accelerates the degradation and recycling of macromolecules, thereby providing essential nutrients for cancer cell growth [11].

Chloride channels, particularly chloride channel 3 (CLC3), have emerged as key lysosomal acidification and function regulators. CLC, a member of the CLC family of chloride channels and transporters, regulates ionic homeostasis across cellular membranes [12]. CLC3 is primarily localized to intracellular compartments, including lysosomes, where it facilitates the exchange of chloride ions and protons, maintaining the electrochemical gradient necessary for V-ATPase activity and lysosomal acidification. Recent studies have implicated CLC3 in various physiological and pathological processes, such as cell proliferation and apoptosis [13, 14]. Additionally, its role in malignant glial cells has attracted attention, with evidence suggesting that CLC3 activation is involved in glioma progression and may contribute to tumor growth and resistance to therapy [15, 16]. Additionally, studies have reported that CLC3 induces paclitaxel resistance in ovarian cancer cells [17]. Similarly, our previous work confirmed high CLC3 expression in cervical cancer and demonstrated that inhibiting ClC-3 expression enhances the sensitivity of cervical cancer to cisplatin [18]. However, the mechanism by which CLC3-mediated lysosomal acidification contributes to cervical cancer progression remains unclear.

In the context of CVC, the role of CLC3 remains largely unexplored. Given the critical role of lysosomal function in cancer cell survival and chemoresistance, understanding how CLC3 regulates lysosomal acidification and its impact on CVC progression could provide valuable insights into new therapeutic strategies. Specifically, targeting CLC3 and its regulatory effects on V-ATPase could disrupt the lysosomal functions that are vital for cancer cell survival under chemotherapy, thereby enhancing the efficacy of existing treatments. This study aims to investigate the role of CLC3 in CVC, focusing on its regulation of lysosomal acidification, protein degradation, and chemoresistance.

## Method

### Cell culture and transfection

CVC cell lines (SiHa, MS751, HeLa, ME180, Hela229, CaSKi) and normal cervical epithelial cells (NCCs) were obtained from the American Type Culture Collection (ATCC) and maintained in Dulbecco’s Modified Eagle Medium (DMEM) supplemented with 10% fetal bovine serum (FBS) and 1% penicillin-streptomycin at 37 °C in a humidified atmosphere with 5% CO2. Cells were cultured in T75 flasks and passaged every 3–4 days using trypsin-EDTA. All cell experiments were performed in triplicate.

For siRNA-mediated knockdown experiments, cells were seeded in 6-well plates at a density of 2 × 10^5^ cells per well and allowed to adhere overnight. Transient transfections were performed using Lipofectamine RNAiMAX (Invitrogen) according to the manufacturer’s instructions. Briefly, CLC3-specific siRNA (siCLC3) or non-targeting control siRNA (siControl) was diluted in Opti-MEM reduced serum medium and mixed with Lipofectamine RNAiMAX reagent. The siRNA-Lipofectamine complexes were added to cells in an antibiotic-free growth medium and incubated for 6 h, followed by replacement with a complete growth medium.

### Western blot analysis

Cells were harvested 48 h post-transfection and washed twice with ice-cold phosphate-buffered saline (PBS). Total protein was extracted using RIPA buffer (Thermo Fisher Scientific) supplemented with protease and phosphatase inhibitors (Roche). Protein concentrations were determined using the Pierce BCA Protein Assay Kit (Thermo Fisher Scientific). Equal amounts of protein (20–30 μg) were separated by SDS-PAGE using 8–12% polyacrylamide gels and transferred onto PVDF membranes (Millipore). Membranes were blocked with 5% non-fat milk in Tris-buffered saline containing 0.1% Tween-20 (TBST) for 1 h at room temperature and probed overnight at 4 °C with primary antibodies against CLC3 (1:1000, Abcam), p62 (1:1000, Abcam), LC3Ⅱ/LC3Ⅰ (1:2000, Abcam), Cleaveed-Caspase 3/8 (1:1000, Cell Signaling), and β-actin (1:5000, Sigma-Aldrich) as a loading control. After washing with TBST, membranes were incubated with horseradish peroxidase-conjugated secondary antibodies (1:5000, Santa Cruz Biotechnology) for 1 h at room temperature. Protein bands were visualized using enhanced chemiluminescence substrate (Thermo Fisher Scientific) and captured on X-ray film or using a ChemiDoc Imaging System (Bio-Rad).

### qRT-PCR

Total RNA was extracted from CVC cell lines (SiHa, MS751, HeLa, ME180, Hela229, CaSKi) and normal cervical epithelial cells (NCCs) using TRIzol reagent, and cDNA was synthesized. Real-time PCR was performed with specific primers for CLC3. The gene expression was normalized to GAPDH and quantified using the 2^(-ΔΔCt) method, on a QuantStudio 7 Flex Real-Time PCR System (Applied Biosystems).

### pH measurement of cytosol and lysosomes

Cytoplasmic pH was measured using SypHer3s, Lysosomal pH was measured using LysoSensor Yellow/Blue DND-160. SiHa and CaSKi CVC cells were seeded in glass-bottom dishes or on coverslips in 6-well plates and cultured overnight at 37°C with 5% CO₂ to allow adherence. Cells were incubated with the SypHer3s probe for cytosolic pH measurement, and with LysoSensor Yellow/Blue DND-160 probe for lysosomal pH measurement, respectively, for 30 min at 37 °C in HBSS buffer. After incubation, cells were rinsed twice to remove excess probe. For cytosolic pH measurement, fluorescence images were acquired using a microscope with filters for Ex = 490 nm and Em = 510 nm to detect cytosolic pH changes. For lysosomal pH measurement, fluorescence imaging was conducted using excitation/emission wavelengths of Ex = 510 nm / Em = 528 nm for yellow and Ex = 370 nm / Em = 440 nm for blue fluorescence.

### Fluorescence detection

Cells were seeded in 24-well plates with glass coverslips, transfected with siRNAs, and incubated with respective probes according to the manufacturer’s instructions. After incubation, cells were washed with PBS, fixed, and mounted onto glass slides. Fluorescence images were acquired using appropriate filter sets on a fluorescence microscope and analyzed using ImageJ software. Changes in lysosomal pH were quantified based on fluorescence intensity ratios.

### Cell proliferation assay

Cell proliferation was assessed using MTT assay. Briefly, siRNA-transfected cells were seeded in 96-well plates at a density of 5 × 10^3^ cells per well, culturing for 24, 48, and 72 h. At each time point, cells were incubated with MTT solution at 37 °C. Formazan crystals were solubilized, and absorbance was measured at 570 nm using a microplate reader (BioTek Instruments).

### Colony formation assay

To evaluate the effect of CLC3 modulation on cell proliferative capacity, a colony formation assay was conducted using SiHa and CaSKi CVC cells. Cells from the control, CLC3 overexpression (CLC3^High^), CLC3 knockdown (CLC3^Low^), and a2v-mAb-treated groups were seeded in 6-well plates at a density of 500 cells per well. Cells were incubated at 37 °C with 5% CO₂ for 14 days to allow colony formation. After incubation, colonies were rinsed with PBS, fixed with 4% paraformaldehyde for 20 min, and stained with 0.1% crystal violet for 20 min. Excess dye was washed off, and plates were air-dried. Colonies were visualized and photographed under a microscope.

### Apoptosis analysis

Flow cytometric analysis was performed. Briefly, cells were harvested 48 h post-transfection, washed with PBS, and fixed in ice-cold 70% ethanol overnight at −20 °C. Fixed cells were stained with propidium iodide (PI) solution containing RNase A and analyzed using a BD FACSCalibur flow cytometer (BD Biosciences). Cell cycle distribution and apoptotic cell populations (sub-G1 phase) were quantified using FlowJo software (Tree Star).

### In vivo tumor xenograft model

Animal experiments were conducted in accordance with institutional guidelines and approved by the Institutional Animal Care and Use Committee (IACUC). Animal studies were approved by the Institutional Animal Care and Use Committee of [ZJCLA-ACUC-20010745]. Female athymic nude mice (4–6 weeks old) were obtained from Charles River Laboratories and housed under specific pathogen-free conditions with access to food and water. The experimental mice were randomly divided into 4 groups, with 6 mice in each group. Sequentially, these were the control group, cisplatin group, CLC3 knockdown combined with cisplatin group, and CLC3 overexpression combined with cisplatin group. HeLa cells transfected with siCLC3 or CLC3 were subcutaneously injected into the right flank of mice. Cisplatin was administered via intraperitoneal injection (3 mg/kg cisplatin dissolved in 100 μL saline). The blank control group received an intraperitoneal injection of 100 μL saline.

Tumor growth was monitored by measuring perpendicular tumor diameters using calipers every 3 days. Tumor volume was calculated using the formula:

$${\rm{Volume}}=\frac{{Length}\times {{Width}}^{2}}{2}.$$

At the end of the experiment, mice were sacrificed, and tumors were collected for histological analysis. From cell line injection to the completion of all analyses, the researchers remained blinded.

### TUNEL assay

Tumor tissues were fixed in 10% formalin and embedded in paraffin. A TUNEL assay was performed using the In Situ Cell Death Detection Kit (Roche) for detecting apoptotic cells in tumor sections.

### Immunohistochemistry (IHC)

For IHC, tissue sections were deparaffinized, rehydrated, and subjected to antigen retrieval using citrate buffer (pH 6.0). Endogenous peroxidase activity was blocked with 3% hydrogen peroxide, and sections were incubated with primary antibodies against CLC3 (1:100, Abcam), cyclin D1 (1:100, Abcam), cyclin D3 (1:800, Cell Signaling) and Ki-67 (1:500, Abcam) overnight at 4 °C. After washing, sections were incubated with horseradish peroxidase-conjugated secondary antibodies, developed using DAB substrate, and counterstained. Stained sections were examined under a light microscope, and images were captured for quantitative analysis.

### Lung metastases assay

To assess the impact of CLC3 modulation on lung metastases of CVC cells, an in vivo lung metastases model was established. The experimental mice were randomly divided into 4 groups, with 6 mice in each group. CaSKi cells from different treatment groups (control, CLC3^High^ combined with cisplatin treatment, CLC3^low^ combined with cisplatin treatment, cisplatin treatment alone) were injected into the tail veins of immunodeficient mice to simulate metastatic spread to the lungs. Four weeks post-injection, mice were sacrificed, and lung tissues were collected and processed hematoxylin and eosin (H&E). The number of metastatic nodules in each lung was counted under a microscope to evaluate the metastatic potential of CVC cells in each group.

### Cell viability and IC50 determination (MTT assay)

To determine cell viability and IC50 values, siRNA-transfected cells were seeded in 96-well plates at a density of 5 × 10^3^ cells per well and treated with increasing concentrations of anticancer drugs or compounds targeting lysosomal function. After 48 h of treatment, cell viability was assessed using the MTT assay as described previously. IC50 values were calculated using GraphPad Prism software based on dose-response curves generated from triplicate experiments.

### RNA extraction and real-time PCR (RT-PCR)

Total RNA was extracted from siRNA-transfected CVC cells using TRIzol reagent, and its concentration and purity were assessed using a NanoDrop spectrophotometer. Complementary DNA (cDNA) was synthesized from 1 μg of RNA using the SuperScript IV Reverse Transcriptase Kit with random hexamers. RT-PCR was performed using specific primers for V-ATPase subunits (ATP6V1A, ATP6V1B1, ATP6V1B2, ATP6V1C1, ATP6V1C2, ATP6V1C3, ATP6V1D, ATP6V1E1, ATP6V1E2, ATP6V1F, ATP6V1G1, ATP6V1G2, ATP6V1G3, ATP6V1H, ATP6V0A1, ATP6V0A2, TCIRG1, ATP6V0A4, ATP6V0C, ATP6V0B, ATP6V0D1, ATP6V0D2, ATP6V0E1, ATP6V0E2, RNASEK, ATP6AP1, ATP6AP2, TMEM199, VMA21 and CCDC115), with amplification on a StepOnePlus Real-Time PCR System.

Primers specific for V-ATPase subunits were designed using Primer-BLAST (NCBI) and validated for specificity and efficiency. Relative mRNA expression levels were quantified using the 2 − ΔΔCt method, normalized to GAPDH expression, and presented as fold change compared to siControl-treated cells.

### DQ-BSA assay

To assess lysosomal proteolytic activity, the DQ™ Green BSA assay (Thermo Fisher Scientific, D12050) was performed. Briefly, cervical cancer cells (control, CLC3^high^, and CLC3^low^) were seeded on coverslips in 24-well plates and incubated overnight. Cells were then incubated with 10 μg/mL DQ-Green BSA diluted in serum-free medium at 37 °C for 2 h. After washing with PBS, cells were fixed with 4% paraformaldehyde and mounted using anti-fade medium. Fluorescence intensity was visualized using a fluorescence microscope, and images were quantified with ImageJ software.

### Annexin V/PI staining

To evaluate apoptosis and investigate its potential relationship with autophagy, Annexin V/propidium iodide (PI) double staining was performed using the Annexin V-FITC/PI Apoptosis Detection Kit (BD Biosciences, USA) according to the manufacturer’s instructions. Briefly, cervical cancer cells (control, CLC3^low^, CLC3^high^) were harvested, washed with cold PBS, and resuspended in binding buffer. Annexin V-FITC and PI were added, followed by incubation in the dark for 15 min at room temperature. Fluorescence signals were detected using a flow cytometer (BD FACSCalibur), and apoptotic populations were quantified. To explore the relationship between autophagy and apoptosis, Bafilomycin A1 (BafA1, 10 nM) was applied to CLC3-knockdown cells to inhibit autophagy, while rapamycin (50 nM) was used to induce autophagy in CLC3-overexpressing cells for 12 h before staining.

### Calcein-AM/PI cell viability staining

Cell viability was assessed using Calcein-AM and propidium iodide (PI) staining. Live cells with intact membranes enzymatically convert non-fluorescent Calcein-AM into green-fluorescent calcein, while PI is a red-fluorescent dye that only enters cells with compromised membranes, labeling dead cells. The stained cells were analyzed by flow cytometry.

### Co-immunoprecipitation

Co-IP assays were performed to investigate the potential interaction between CLC3 and the V-ATPase subunit ATP6V1A. Cervical cancer cells were lysed in ice-cold RIPA buffer (Thermo Scientific) supplemented with protease and phosphatase inhibitors. The lysates were centrifuged to remove debris, and the supernatants were collected. Protein concentrations were determined using a BCA Protein Assay Kit (Thermo Fisher Scientific). For immunoprecipitation, equal amounts of total protein (500–800 μg) were incubated overnight at 4 °C with 2–3 μg of anti-CLC3 antibody or normal rabbit IgG as a negative control, followed by incubation with protein A/G agarose beads (Santa Cruz Biotechnology). Beads were then washed five times with RIPA buffer, and bound proteins were eluted by boiling in SDS loading buffer for 5 min. The immunoprecipitates were separated by SDS-PAGE and subjected to Western blotting using antibodies against CLC3.

### Sphere formation assay

To evaluate the effect of CLC3 expression on tumor stemness, sphere formation assays were performed. Cervical cancer cells with differential CLC3 expressions (control, CLC3^low^, CLC3^high^) were treated with a2v-mAb and seeded in ultra-low attachment 6-well plates at a density of 1000 cells/well in serum-free DMEM/F12 medium supplemented with 20 ng/mL EGF, 20 ng/mL bFGF, and B27 supplement. After 7–10 days of culture, tumor spheres larger than 50 μm were counted under an inverted microscope. The number of spheres formed was used as an indicator of self-renewal capacity.

### Statistical analysis

All experiments were performed in triplicate. Data are presented as mean ± standard deviation. Statistical evaluation using Student’s *t*-test or one-way analysis of variance (ANOVA). *P*-values less than 0.05 were considered statistically significant.

## Results

### CLC3 modulation alters chemosensitivity in CVC cells

**Fig. 1:**
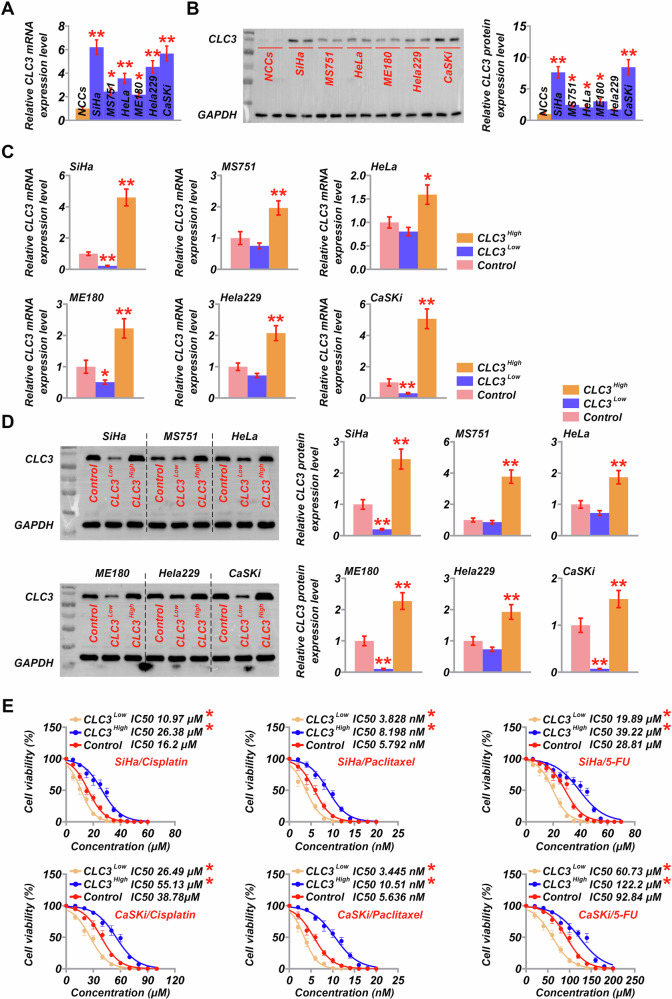
Analysis of CLC3 expression in cervical cancer cell lines and its impact on chemoresistance. **A** Relative CLC3 mRNA expression levels in various CVC cell lines (SiHa, MS751, HeLa, ME180, Hela229, CaSKi) compared to normal cervical epithelial cells (NCCs). *P < 0.05, **P < 0.001. vs. NCCs. **B** Western blot analysis showing CLC3 protein expression in NCCs and different CVC cell lines (SiHa, MS751, HeLa, ME180, Hela229, CaSKi), with GAPDH as a loading control. *P < 0.05, **P < 0.001. vs. NCCs. **C** CLC3 mRNA expression in CVC cell lines (SiHa, MS751, HeLa, ME180, Hela229, and CaSKi) following CLC3 overexpression (CLC3^High^) or knockdown (CLC3^Low^) compared to control. *P < 0.05, **P < 0.001. vs. Control. **D** Western blot analysis of CLC3 protein levels in CLC3^High^, CLC3^Low^, and control cells across the same cell lines, with quantification shown in the accompanying graphs. *P < 0.05, **P < 0.001. vs. Control. **E** IC50 values for cisplatin, paclitaxel, and 5-FU in CLC3^High^, CLC3^Low^, and control groups in SiHa and CaSKi cells, showing CLC3 modulation’s effect on chemoresistance. *P < 0.05 vs. Control.

The expression of CLC3 was evaluated across several human CVC cell lines and normal cervical epithelial cells. The results from PCR and Western blot analysis indicated that CLC3 expression was significantly elevated in all CVC cell lines relative to NCCs, with the highest levels observed in SiHa and CaSKi cells (Fig. 1A, B). Specifically, the expression levels in SiHa and CaSKi were approximately fourfold higher than in NCCs, indicating a potential role for CLC3 in CVC progression. We then downregulated and overexpressed CLC3 in these human cancer cell lines to confirm transfection efficiency. SiHa and CaSKi cells, which showed more pronounced effects during transfection, were selected to assess the impact of CLC3 on chemosensitivity (Fig. 1C, D). Transfected SiHa and CaSKi cells were treated with 5-fluorouracil (5-FU), cisplatin, and paclitaxel. Cell viability and IC50 calculations revealed that CLC3 downregulation significantly increased the sensitivity of SiHa and CaSKi cells to all three chemotherapeutic agents. Compared with the control SiHa cells, CLC3 knockdown significantly reduced the IC₅₀ values for cisplatin, Pa, and 5-FU (*P* = 0.0311, 0.0428, 0.0429, respectively; Fig. 1E). Conversely, CLC3 overexpression resulted in reduced sensitivity to these drugs, with increased IC₅₀ values (*P* = 0.0332, 0.0235, 0.0385). In CaSKi cells, CLC3 knockdown similarly reduced IC₅₀ values for Cisplatin, Pa, and 5-FU (*P* = 0.0421, 0.0429, 0.0242, Fig. 1E). Overexpression of CLC3 reduced sensitivity to these drugs, increasing the IC50 values (*P* = 0.0377, 0.0196, 0.0452, Fig. 1E), indicating that CLC3 overexpression confers chemoresistance to CVC cells.

### CLC3 enhances lysosomal acidification and protein degradation

**Fig. 2:**
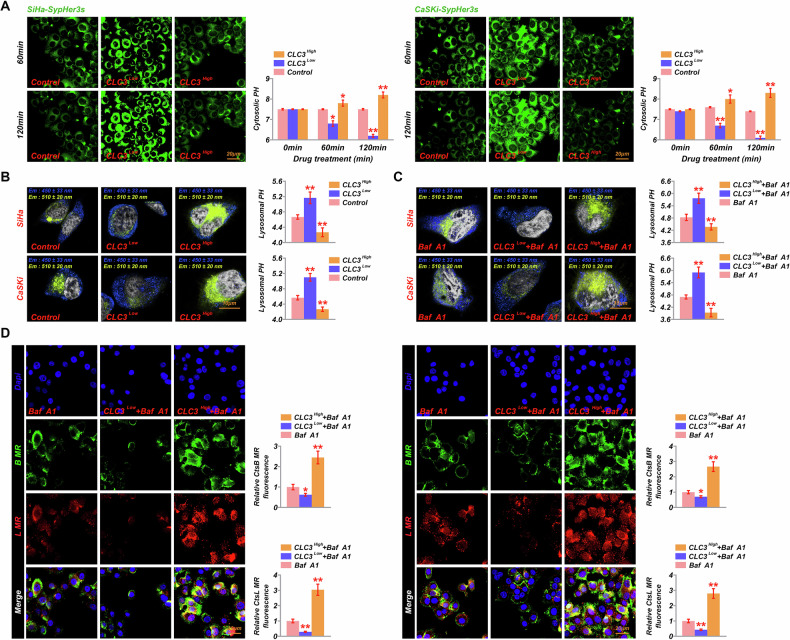
Effect of CLC3 modulation on lysosomal pH and protein degradation in cervical cancer cells. **A** Fluorescence images and bar graphs showing cytosolic pH in SiHa and CaSKi cells with CLC3 overexpression (CLC3^High^), knockdown (CLC3^Low^), and control, using SypHer3s as the pH probe at 60- and 120-min post-treatment. **B** Representative images and quantification of lysosomal pH in SiHa and CaSKi cells with CLC3 modulation, analyzed using LysoSensor Yellow/Blue DND-160. **C** Lysosomal pH in cells treated with V-ATPase inhibitor Baf A1 combined with CLC3^High^ and CLC3^Low^ modulation. **D** Immunofluorescence images showing the fluorescence intensity of cathepsin B (B MR) and cathepsin L (L MR) in cells treated with Baf A1 and CLC3-modulated conditions, indicating lysosomal degradation capacity. *P < 0.05, **P < 0.001. vs. Control.

The role of CLC3 in cytoplasm and lysosomes was examined using the SypHer3s probe and LysoSensor Yellow/Blue DND-160. After 120 min of incubation, fluorescence microscopy revealed that increased CLC3 expression promoted cytoplasmic alkalization, while CLC3 downregulation led to a significant opposite effect (Fig. 2A). In CLC3-upregulated cells, a considerable increase in the yellow/blue fluorescence ratio was observed, indicating enhanced lysosomal acidification compared to control cells. Conversely, CLC3-downregulated cells exhibited reduced acidification, with a shift toward a more neutral lysosomal pH (Fig. 2B). These findings were further supported by the quantification of lysosomal pH values, which showed that CLC3-overexpressing cells maintained a significantly lower pH, thereby creating optimal conditions for lysosomal enzyme activity. Additionally, when V-ATPase activity was inhibited by Bafilomycin A1 (Baf A1), cells overexpressing CLC3 maintained significantly lower lysosomal pH even after Baf A1 treatment (Fig. 2C), suggesting that CLC3 may partially compensate for V-ATPase inhibition. Moreover, we observed only a modest increase in lysosomal pH after Baf A1 treatment in control cells. This attenuated response may be due to the high basal expression of CLC3 in these cells, which could sustain proton influx and preserve lysosomal acidity despite V-ATPase blockade (Fig. S1A). Moreover, fluorescence detection of lysosomal cathepsin B and L (using B MR and L MR markers) showed that protein degradation activity was highest in CLC3-upregulated cells, while CLC3-downregulated cells exhibited diminished lysosomal protease activity (Fig. 2D). To further confirm the impact of CLC3 on lysosomal degradation, a DQ-BSA assay was conducted. The results showed that CLC3-overexpressing cells displayed significantly higher green fluorescence intensity compared to control cells, indicating enhanced lysosomal proteolytic activity. Conversely, CLC3-knockdown cells exhibited diminished fluorescence, suggesting reduced lysosomal degradation capacity (Fig. S1B). This suggests that CLC3 may not only promote lysosomal acidification but also enhances lysosomal degradation capacity.

### CLC3 deficiency enhances autophagy and apoptosis

**Fig. 3:**
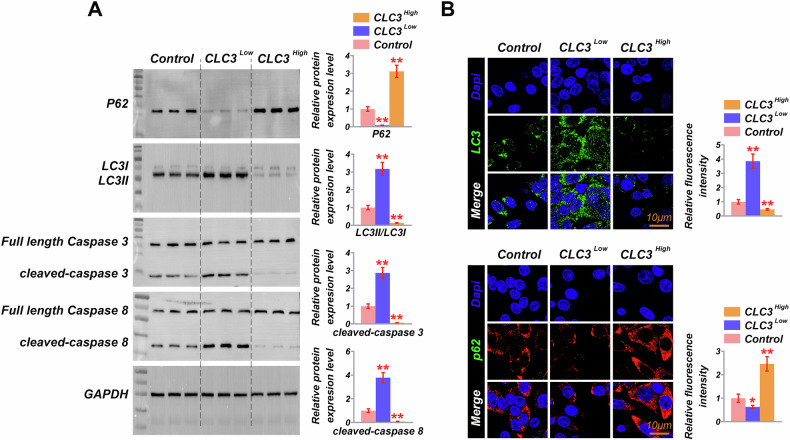
Effects of CLC3 modulation on autophagy and apoptosis in cervical cancer cells. **A** Western blot analysis of autophagy and apoptosis markers, including P62, LC3 (LC3I and LC3II), and cleaved caspases-3 and -8 in CLC3^High^, CLC3^Low^, and control cells. Bar graphs quantify the relative expression levels of these proteins, showing increased autophagic flux and apoptotic activity in CLC3^Low^ cells. **B** Immunofluorescence staining of LC3 and P62 in CLC3-modulated cells, with quantification of relative fluorescence intensity in CLC3^High^, CLC3^Low^, and control groups. *P < 0.05, **P < 0.001. vs. Control. Data are presented as mean ± SD. Statistical significance was determined using one-way ANOVA with post hoc tests; *p* < 0.05, *p* < 0.01, *p* < 0.001.

Western blot analysis was employed to examine the levels of autophagy and apoptosis-related proteins in SiHa and CaSKi cells with altered CLC3 expression (Fig. 3). In CLC3-downregulated cells, there was a significant decrease in P62 levels, indicating enhanced autophagic flux. This was further supported by an increased LC3II/LC3I ratio, suggesting an upregulation of autophagy. In contrast, CLC3-upregulated cells exhibited significantly elevated P62 levels and a significantly decreased LC3II/LC3I ratio, indicating a suppression of autophagy. Regarding apoptosis, CLC3-downregulated cells showed significantly elevated levels of cleaved caspase-3 and caspase-8, indicating an increase in apoptotic activity. Conversely, CLC3-upregulated cells demonstrated reduced levels of these cleaved caspases, suggesting that CLC3 overexpression inhibits apoptosis (Fig. 3A). Further immunofluorescence staining of LC3 and P62 experiments prove those findings (Fig. 3B). Calcein-AM/PI and Annexin V/PI staining revealed that CLC3 knockdown significantly increased apoptotic cell populations compared to control cells. Treatment with BafA1, an autophagy inhibitor, did not attenuate this apoptotic increase in CLC3-deficient cells, suggesting that the induction of apoptosis in this context occurs independently of autophagy. Conversely, CLC3 overexpression reduced the proportion of apoptotic cells, and treatment with rapamycin, an autophagy inducer, did not reverse this anti-apoptotic effect (Fig. S1C–D). These results suggest that CLC3 deficiency enhances both autophagy and apoptosis independently in CVC cells, while CLC3 overexpression suppresses these processes, potentially contributing to increased cell survival and chemoresistance.

### CLC3 regulates lysosomal function through V-ATPase modulation

**Fig. 4:**
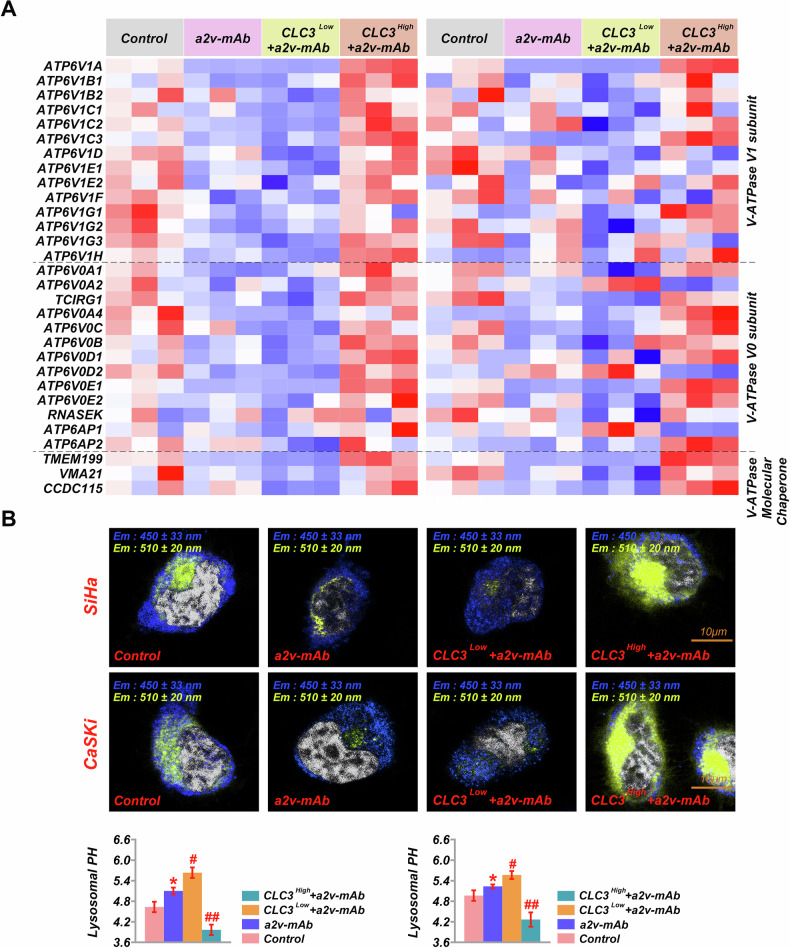
Impact of V-ATPase inhibition and CLC3 modulation on V-ATPase subunit expression and lysosomal pH. **A** Heatmap showing the expression of various V-ATPase subunits and companion genes across control, a2v-mAb-treated, CLC3^Low^ with a2v-mAb, and CLC3^High^ with a2v-mAb groups in CVC cells. **B** Lysosomal pH analysis in SiHa and CaSKi cells under the same experimental conditions, with images from LysoSensor Yellow/Blue DND-160 assays and quantitative analysis of pH changes. *P < 0.05, **P < 0.001. vs. Control. ^#^*P* < 0.05, ^##^*P* < 0.001. vs. a2v-mAb.

The observation that ‘CLC3 overexpression compensates for V-ATPase inhibition by further enhancing acidification’ suggests a potential interaction between CLC3 and V-ATPase. RT-PCR analysis indicated that a2v-mAb treatment downregulated several V-ATPase subunits and associated genes, particularly ATP6V1A, in both control and CLC3-modified cells. Notably, ATP6V1A was significantly upregulated in CLC3-overexpressing cell (Fig. 4A). To further investigate a physical interaction, we performed co-immunoprecipitation (Co-IP) assays. Western blotting of anti-CLC3 immunoprecipitates revealed that ATP6V1A was specifically co-precipitated (Fig. S1E), and the reciprocal Co-IP confirmed the presence of CLC3 in ATP6V1A pull-downs. Functionally, V-ATPase inhibition by a2v-mAb reduced lysosomal acidification, as visualized using LysoSensor Yellow/Blue DND-160 staining in both control and CLC3-knockdown SiHa and CaSKi cells (Fig. 4B). CLC3-knockdown cells exhibited a more pronounced loss of acidification, indicating an impaired acidification mechanism. In contrast, CLC3-overexpressing cells maintained low lysosomal pH even after V-ATPase inhibition, indicating that CLC3 may partially compensate for reduced V-ATPase activity. These results recommend that CLC3 is crucial in modulating lysosomal pH, potentially by interacting with V-ATPase components and influencing their activity

### CLC3 modulates tumor stemness and inhibits apoptosis

**Fig. 5:**
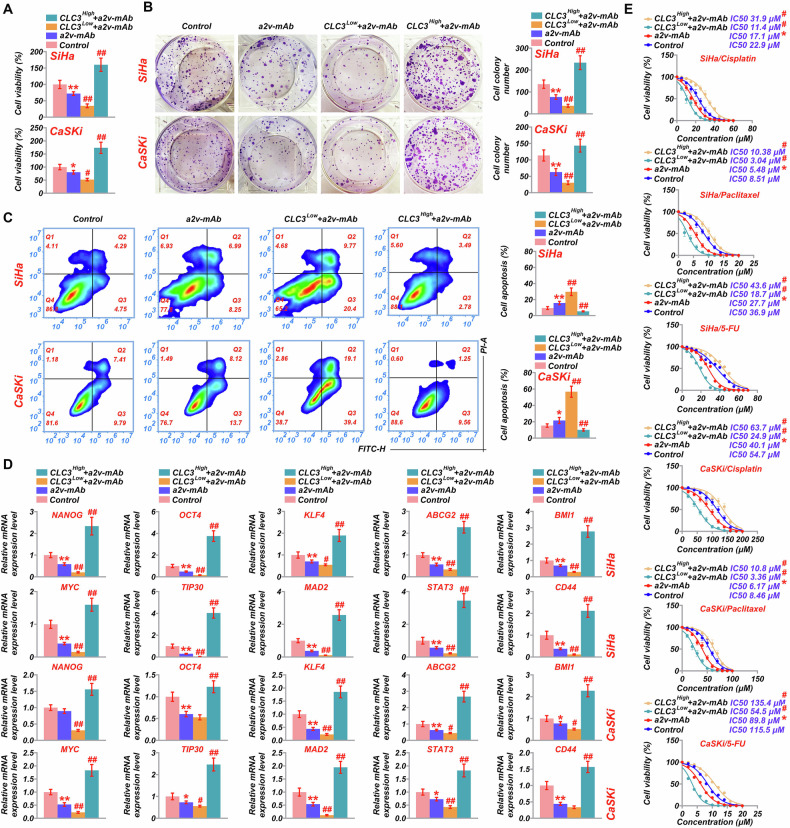
Effects of CLC3 modulation and V-ATPase inhibition on cell proliferation, apoptosis, and tumor stemness markers. **A** Cell viability assays in SiHa and CaSKi cells under various treatment conditions, including a2v-mAb treatment, CLC3^High^, and CLC3^Low^. **B** Colony formation assay results, quantifying cell colony numbers to evaluate the proliferative capacity of each treatment group. **C** Flow cytometry analysis of apoptosis rates in SiHa and CaSKi cells with CLC3 modulation and a2v-mAb treatment, with quantification of apoptotic cells. **D** RT-PCR analysis of tumor stemness markers (NANOG, OCT4, KLF4, ABCG2, BMI1, MYC, TIP30, MAD2, STAT3) and CLC3 expression levels in CLC3-modulated cells treated with a2v-mAb, demonstrating the effect of CLC3 on maintaining stemness characteristics. **E** IC50 values for cisplatin, paclitaxel, and 5-FU in SiHa and CaSKi cells under each treatment condition. *P < 0.05 vs. Control. ^#^*P* < 0.05 vs. a2v-mAb.

A series of assays was performed to explore the effects of CLC3 on tumor stemness and related cellular processes. Cell proliferation assays showed that CLC3-upregulated cells treated with a2v-mAb had significantly higher cell viability compared to the a2v-mAb group (Fig. 5A). Conversely, the a2v-mAb-treated group displayed decreased cell viability compared to the control group, and CLC3-downregulated cells treated with a2v-mAb displayed even lower cell viability than the a2v-mAb group, indicating that CLC3 overexpression can partially compensate for the inhibitory effects of V-ATPase inhibition on cell growth. Cell colony formation analysis mirrored these findings, with CLC3-upregulated cells in the presence of a2v-mAb showing a significantly higher cell colony number. In contrast, CLC3-downregulated cells treated with a2v-mAb had the lowest cell colony number, suggesting that CLC3 overexpression accelerates cell cycle progression and compensates for the inhibition of V-ATPase (Fig. 5B). Apoptosis assays further supported these observations. CLC3-upregulated cells treated with a2v-mAb exhibited lower apoptosis rates compared to CLC3-downregulated and control cells, indicating that CLC3 modulates V-ATPase activity to enhance cell survival and support tumor growth (Fig. 5C). To assess the impact of CLC3 on tumor stemness, PCR analysis was performed to measure the expression of key stemness markers, including NANOG, OCT4, and KLF4. CLC3-upregulated cells treated with a2v-mAb exhibited significantly higher expression levels of these markers than those in the a2v-mAb group. In contrast, CLC3-downregulated SiHa cells treated with a2v-mAb showed decreased expression levels, underscoring the potential role of CLC3 in enhancing tumor stemness by modulating V-ATPase (Fig. 5D).

Sphere formation analysis revealed that CLC3 overexpression significantly enhanced the ability of cervical cancer cells to form spheres in the presence of a2v-mAb, while CLC3 knockdown markedly impaired sphere-forming capacity (Fig. S1F). These findings suggest that CLC3 promotes tumor stemness, potentially by modulating lysosomal function through the regulation of V-ATPase activity. Furthermore, IC50 calculations from cell proliferation assays revealed that CLC3-downregulated cells treated with the a2v monoclonal antibody exhibited enhanced sensitivity to all three tested chemotherapeutic agents (Fig. 5E). Compared to SiHa cells, the addition of a2v-mab amplified sensitivity to cisplatin, Pa, and 5-FU (*P* = 0.0405, 0.0312, 0.0416, Fig. 5E), while the addition of a2v-mab in CLC3-knockdown SiHa cells exhibited even greater sensitivity (*P* = 0.0421, 0.0365, 0.0297). Conversely, a2v-mAb’s effects on cisplatin, Pa, and 5-FU were counteracted in CLC3-overexpressing SiHa cells (*P* = 0.0196, 0.0204, 0.0361). Compared to CaSKi cells, the addition of a2v-mAb similarly enhanced sensitivity to cisplatin, Pa, and 5-FU (*P* = 0.0309, 0.0455, 0.0408, Fig. 5E). In CLC3-knockdown SiHa cells, a2v-mAb administration resulted in further enhanced sensitivity (*P* = 0.0164, 0.0378, 0.0257). Conversely, CLC3 overexpression in SiHa cells conferred greater resistance to these chemotherapeutic agents (*P* = 0.0257, 0.0265, 0.0244). This increased resistance, along with enhanced tumor stemness and elevated cell proliferation, highlights the role of CLC3 in tumor growth and survival.

### CLC3 depletion increases cisplatin sensitivity and inhibits tumor growth in vivo

**Fig. 6:**
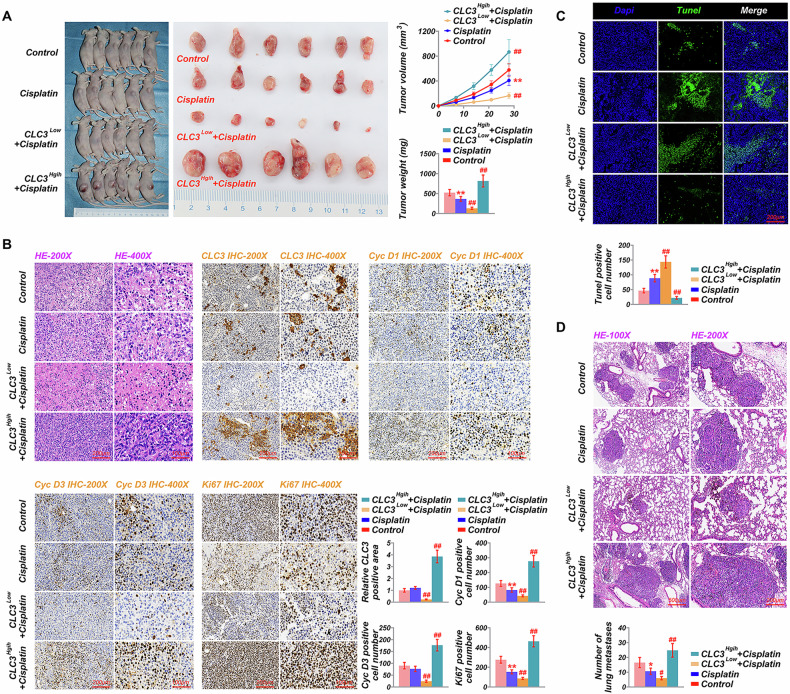
In vivo effects of CLC3 modulation on tumor growth, metastases, and response to cisplatin in a mouse xenograft model. **A** Tumor volume and weight measurements in mice treated with cisplatin, CLC3^Low^ with cisplatin, CLC3^High^ with cisplatin, and control. **B** Hematoxylin and eosin (H&E) staining, along with immunohistochemical (IHC) staining for CLC3, cyclin D1, cyclin D3, and Ki67 in tumor sections from each group, with quantification of positive cells. **C** TUNEL assay showing apoptosis levels in tumors from each treatment condition, with quantification of TUNEL-positive cells. **D** Histological analysis of lung tissue for metastases, comparing the number of metastatic lung nodules across different treatment groups, highlighting the anti-metastatic effect of CLC3 knockdown with cisplatin. *P < 0.05, **P < 0.001. vs. Control. ^#^*P* < 0.05, ^##^*P* < 0.001. vs. Cisplatin.

A mouse xenograft model was used to investigate the effects of CLC3 modulation on tumor growth and response to cisplatin treatment. CLC3 knockdown, combined with cisplatin treatment, led to a significant reduction in tumor volume compared to the control and cisplatin-alone groups, indicating that CLC3 downregulation enhances sensitivity to cisplatin (Fig. 6A). TUNEL assays confirmed significantly increased apoptosis in the CLC3 knockdown + cisplatin group, further supporting the enhanced chemosensitivity conferred by CLC3 depletion (Fig. 6C). Immunohistochemical analysis revealed a marked reduction in proliferation markers, including cyclin D1, cyclin D3, and Ki-67, as well as decreased CLC3 expression in this group (Fig. 6B). Additionally, lung metastases assays showed the most pronounced inhibition of lung metastases in the CLC3 knockdown and cisplatin-treated group (Fig. 6D). These findings suggest that CLC3 knockdown sensitizes CVC cells to cisplatin, impedes tumor progression, and effectively inhibits lung metastases. In contrast, tumors overexpressing CLC3 exhibited larger volumes than all other groups, even with cisplatin treatment, indicating that CLC3 overexpression confers resistance to cisplatin (Fig. 6A). TUNEL assay results showed reduced apoptosis levels in tumors overexpressing CLC3, reflecting decreased chemosensitivity (Fig. 6B). Immunohistochemical analysis further demonstrated elevated levels of proliferation markers and increased CLC3 expression in these tumors (Fig. 6C), while the inhibitory effect on lung metastases in the CLC3 overexpression and cisplatin-treated group was minimal (Fig. 6D). Overall, these results demonstrate that CLC3 overexpression promotes tumor growth and confers resistance to cisplatin, whereas CLC3 depletion enhances chemosensitivity, reduces tumor growth, and inhibits metastases in vivo.

## Discussion

The findings of this study provide compelling evidence that CLC3 plays a critical role in regulating lysosomal function, chemoresistance, and tumorigenic properties in CVC cells. Through a series of experiments, we demonstrated that CLC3 expression is significantly upregulated in CVC cells. This upregulation is closely associated with enhanced cytosolic alkalinization, lysosomal acidification, increased lysosomal protein degradation, inhibition of cell autophagy and apoptosis independently, and the promotion of chemoresistance and tumor stemness. Furthermore, the modulation of V-ATPase activity by CLC3 suggests a mechanistic link between CLC3 expression and lysosomal function, which is crucial for the proliferation and apoptosis of CVC cells.

One of the most striking discoveries is the role of CLC3 in modulating chemosensitivity in CVC cells. Our results show that CLC3 overexpression significantly reduces the sensitivity of CVC cells to commonly used chemotherapeutic agents, including 5-FU, cisplatin, and paclitaxel. Conversely, CLC3 knockdown enhances chemosensitivity, particularly to cisplatin, as evidenced by the lower IC50 values in CLC3 downregulated cells. Additionally, CLC3 depletion sensitized CVC cells to cisplatin and suppressed tumor growth in vivo. These findings align with previous studies suggesting that chloride channels, especially CLC3, play an important role in modulating drug resistance in cancer cells [17, 19, 20]. Similar observations have been made in cholangiocarcinoma and glioma cells, where the suppression of CLC3 increases cisplatin sensitivity by modulating autophagy [21, 22].

The mechanism by which CLC3 confers chemoresistance appears to be multifaceted. One proposed explanation, as noted by Ye et al, is that CLC3-mediated lysosomal acidification creates a favorable environment for cisplatin sequestration and degradation, thereby reducing cisplatin-induced apoptosis [23]. Lysosomes are critical organelles in cellular metabolism and drug processing. In our study, CLC3 overexpression led to cytosolic alkalinization and lysosomal acidification, as demonstrated by increased yellow/blue fluorescence ratios in LysoSensor assays. These pH changes were accompanied by enhanced lysosomal protein degradation, indicating that CLC3 enhances lysosomal function by preserving acidic conditions in cervical cancer (CVC) cells. To further understand the underlying mechanism, we examined the role of V-ATPase—a proton pump critical for lysosomal acidification—and its modulation by CLC3. V-ATPase dysfunction is implicated in various malignancies, promoting lysosomal activity, autophagy, and chemoresistance [24, 25], and contributing to tumor microenvironment acidification and metastatic potential [26, 27]. In ovarian cancer, V-ATPase inhibition has been shown to suppress tumor growth and boost antitumor immunity [28]. Our data demonstrate that inhibition of V-ATPase suppressed lysosomal acidification, consistent with its well-established role in maintaining acidic lysosomal environments. Interestingly, CLC3 overexpression partially restored lysosomal acidification even under V-ATPase-inhibited conditions, as shown by LysoSensor imaging. This suggests that CLC3 may compensate for reduced V-ATPase activity to some extent, thereby sustaining lysosomal pH homeostasis. Furthermore, RT-PCR analysis revealed that CLC3 overexpression upregulated multiple V-ATPase subunits, particularly ATP6V1A, whereas V-ATPase inhibition significantly downregulated these genes in both the control and CLC3-modulated groups. As shown in Fig. 4A, the transcriptional profiles of V-ATPase components, including V1 and V0 subunits, were distinctly influenced by CLC3 expression and a2v-mAb treatment. And Co-IP analysis demonstrated a physical interaction between CLC3 and the V-ATPase subunit ATP6V1A, suggesting that CLC3 may influence lysosomal acidification through direct modulation of V-ATPase function. These findings indicate that CLC3 may enhance V-ATPase function not only by preserving lysosomal pH but also by facilitating the activity of V-ATPase subunits, thereby promoting proton translocation into the lysosomal lumen. Interestingly, even under pharmacological inhibition of V-ATPase (BafA1 or a2v-mAb), CLC3-overexpressing cells retained significant lysosomal acidification. This observation suggests that CLC3 may also regulate lysosomal pH through mechanisms independent of direct V-ATPase activity. One plausible explanation for this is the canonical role of CLC3 as a Cl⁻/H⁺ exchanger in the lysosomal membrane. By exporting chloride ions in exchange for protons, CLC3 helps maintain the electrochemical gradient necessary for proton retention, indirectly sustaining lysosomal acidity even when V-ATPase function is partially inhibited [29, 30]. Taken together, our findings suggest that CLC3 regulates lysosomal acidification and protein degradation through direct interaction with V-ATPase subunits and V-ATPase-independent mechanisms, such as chloride/proton exchange activity. These complementary pathways may collectively enhance lysosomal function, thereby promoting tumor cell survival and chemoresistance in CLC3-overexpressing cervical cancer cells.

Another significant finding is CLC3’s role in promoting tumor progression in CVC cells. Our results showed that CLC3 deficiency enhanced autophagic flux and promoted apoptosis, as indicated by decreased P62 levels, increased LC3II/LC3I ratios, and elevated cleaved caspase-3 and caspase-8 levels. Calcein-AM/PI and Annexin V/PI staining confirmed the increase in apoptotic cells upon CLC3 knockdown. However, the application of autophagy inhibitor BafA1 did not attenuate apoptosis, and autophagy induction via rapamycin failed to restore apoptotic sensitivity in CLC3-overexpressing cells. These findings suggest that autophagy and apoptosis are independently regulated by CLC3. Previous studies have shown that chloride channels, especially CLC3, can be activated by DSF/Cu to induce apoptosis in prostate cancer [31]. Similarly, CLC3 knockdown has been shown to suppress proliferation and metastases in colorectal cancer via the Wnt/β-catenin pathway [32]. Furthermore, CLC3 has been confirmed to promote cell proliferation by modulating cyclin D1 and P27 [33], which aligns with our findings that CLC3 regulates V-ATPase, particularly ATP6V1A, to foster cell proliferation and inhibit apoptosis in CVC cells. Other studies, such as PHY34’s modulation of V-ATPase subunits and CAS interaction leading to autophagy suppression, further support these findings [34]. Additionally, the disulfiram/copper complex inhibits cell proliferation and induces apoptosis by upregulating CLC3 expression [35]. Our data suggest that CLC3 deficiency affects both autophagy and apoptosis by altering lysosomal acidification via the suppression of V-ATPase. By enhancing lysosomal function and independently suppressing excessive autophagy and apoptosis, CLC3 may play a cytoprotective role in CVC cells, enabling their survival under stress conditions, such as chemotherapy or nutrient deprivation.

Notably, although lysosomal degradation constitutes the terminal step of the autophagic process, our findings revealed a seemingly paradoxical effect wherein CLC3 overexpression enhances lysosomal proteolytic activity, as evidenced by elevated cathepsin B/L activity and increased DQ-BSA degradation, yet concurrently inhibits autophagic flux, as indicated by decreased LC3-II/I ratios and p62 accumulation. This suggests that CLC3-mediated lysosomal activation is not necessarily coupled with autophagy. One explanation for this may involve the previously discussed role of CLC3 in promoting lysosomal acidification via V-ATPase modulation, thereby enhancing lysosomal enzymatic activity. However, this activation may selectively support degradation pathways independent of macroautophagy. Furthermore, CLC3 may influence lysosomal function via transcriptional regulation. It has been shown that V-ATPase activity can affect the nuclear localization and activity of TFEB, a master regulator of lysosomal biogenesis. By enhancing V-ATPase-mediated acidification, CLC3 may indirectly promote TFEB activation, leading to the upregulation of lysosomal genes such as LAMP1 and CTSB [36, 37], thereby boosting proteolytic capacity without necessarily inducing autophagy. This uncoupling between autophagic flux and lysosomal degradation highlights the selective regulatory role of CLC3 in sustaining degradative efficiency while restraining excessive autophagic activation, which may benefit tumor cell survival under stress conditions. Moreover, our findings that autophagy inhibition with BafA1 did not suppress apoptosis in CLC3-knockdown cells and that rapamycin-induced autophagy activation failed to trigger apoptosis in CLC3-overexpressing cells further support the notion that CLC3 may independently regulate these pathways. One possible explanation for this is the activation of the mTORC1 signaling pathway. V-ATPase-mediated lysosomal acidification has been shown to recruits and activates mTORC1 on lysosomal membranes, which in turn phosphorylates and inhibits ULK1, a key initiator of autophagy, thereby preventing autophagosome formation [38, 39]. Given our previous findings that CLC3 promotes lysosomal acidification through V-ATPase stabilization, it is plausible that CLC3 indirectly suppresses autophagy initiation via enhanced mTORC1 activation. The observed p62 accumulation supports this notion, as p62 is a well-established marker of impaired autophagic degradation [39]. Collectively, these results suggest that CLC3 exerts differential control over lysosomal degradation and autophagy, promoting lysosomal acidification and catabolic capacity while restraining autophagic machinery, possibly to maintain metabolic homeostasis and protect cells from autophagy-mediated death under chemotherapy stress. and promotes tumor cell survival under stress conditions, such as chemotherapy.

Tumor stemness, characterized by the expression of specific stem cell markers, is associated with increased tumorigenic potential, resistance to therapy, and the ability to sustain tumor growth [40–42]. Our RT-PCR results showed that CLC3-upregulated cells treated with a2v-mAb exhibit significantly higher expression levels of stemness markers than CLC3-downregulated and control cells. Moreover, the sphere formation assay was conducted to reinforce the mRNA data and provide functional evidence that CLC3 enhances tumor stemness. This suggests that CLC3 maintains stemness in CVC cells, likely by modulating V-ATPase activity, particularly ATP6V1A. By enhancing lysosomal acidification and protein degradation, CLC3 provides cancer stem cells with the metabolic flexibility needed to survive and proliferate under adverse conditions.

The findings of this study have important implications for the development of targeted therapies for CVC. Given the role of CLC3 in promoting chemoresistance, lysosomal function, and tumor stemness, targeting CLC3 or its downstream effectors, such as V-ATPase—especially ATP6V1A—may represent a promising therapeutic strategy. Inhibitors targeting both CLC3 and V-ATPase could disrupt lysosomal function and metabolic homeostasis in cancer cells, sensitizing them to chemotherapy and reducing their tumorigenic potential. Moreover, the combination of CLC3 inhibition with cisplatin, could enhance treatment efficacy by overcoming chemoresistance and promoting apoptosis. Our in vivo results support this approach, as CLC3 knockdown combined with cisplatin treatment significantly reduced tumor growth and increased apoptosis compared to cisplatin treatment alone. These findings suggest that CLC3 inhibition could be an effective adjunct therapy to improve outcomes for CVC patients.

In conclusion, this study demonstrates that CLC3 plays a pivotal role in regulating lysosomal function, chemoresistance, and tumorigenic properties in CVC cells. By modulating V-ATPase activity, CLC3 enhances lysosomal acidification and protein degradation, promoting cell survival and proliferation. Additionally, CLC3 contributes to the maintenance of tumor stemness and the development of chemoresistance, making it a potential target for therapeutic intervention.

## Supplementary information

supplementary legends

Figure S1

Original Data

## Data Availability

The authors declare that all data supporting the findings of this study are available within the paper and any raw data can be obtained from the corresponding author upon request.
