# Correction: CLC3 regulates V-ATPase to enhance lysosomal degradation and cisplatin resistance in cervical cancer cells

**DOI:** 10.1038/s41420-026-03008-y

**Published:** 2026-04-14

**Authors:** Chuyun Chen, Fubin Zhang, Jiayi Shen, Qi Zheng, Zhiyun Zhang, Shun Lu, Lixiao Liu, Tianhong Zhu, Yongming Du, Yutao Guan

**Affiliations:** 1 Center for Reproductive Medicine, the First Affiliated Hospital of Ningbo University, Ningbo, Zhejiang Province China; 2 Department of Obstetrics and Gynecology, the First Affiliated Hospital of Ningbo University, Ningbo, Zhejiang Province China; 3 Health Science Center, Ningbo University, Ningbo, Zhejiang Province China

**Keywords:** Cancer, Genetics

Correction to: *Cell Death Discovery* 10.1038/s41420-025-02876-0, published online 03 December 2025

In this article in fig. 5B the present result was the outcome of our preliminary experiments conducted using 24-well plates, which were not the formal experiments. The formal experiments should have been conducted using 6-well plates. Therefore, we replaced the formal experimental results, and this result had no impact on the conclusion of the article.

Incorrect figure
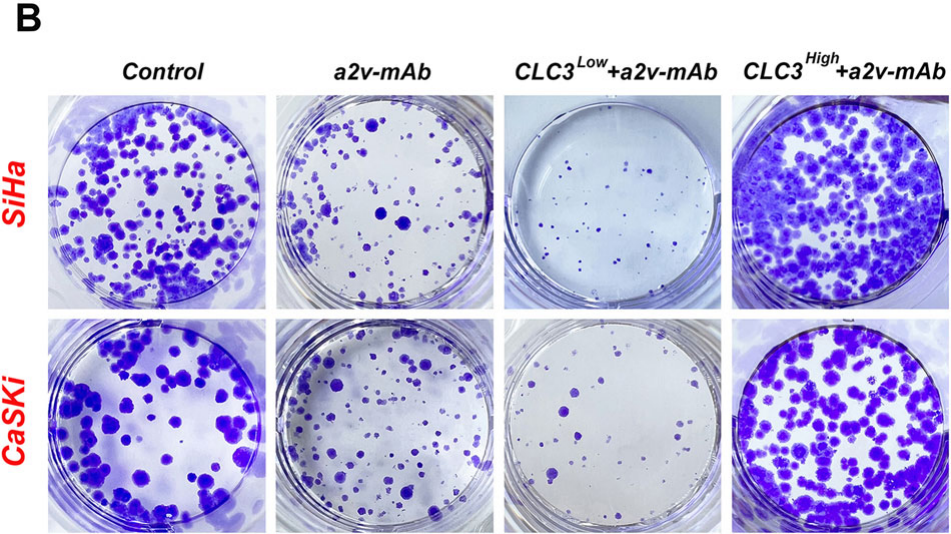


Correct figure
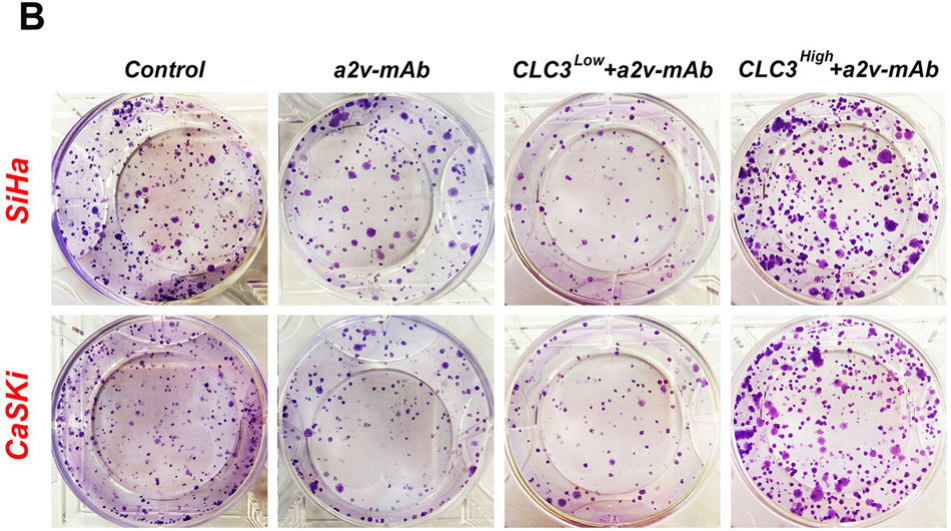


The original article has been corrected.

