# Supplementary material for: CLC3 regulates V-ATPase to enhance lysosomal degradation and cisplatin resistance in cervical cancer cells
Source: Cell Death Discov. 2025 Dec 3;12:5. doi: 10.1038/s41420-025-02876-0 (PMC12783824; doi:10.1038/s41420-025-02876-0)
Supplement: Supplementary file 1 — supplementary legends [file 41420_2025_2876_MOESM1_ESM.doc]

**Supplementary Figure S1**

1. Quantification of lysosomal pH in cervical cancer cells treated with BafA1 or vehicle. *P<0.05, **P<0.001. vs. Control. (B) DQ-BSA assay in cervical cancer cells with altered CLC3 expression compared to control cells. *P<0.05, **P<0.001. vs. Control. (C) Calcein-AM/PI co-staining and (D) Annexin V-FITC/PI staining analysis of control, CLC3^low^, and CLC3^low^ cells treated with BafA1 and control, CLC3^high^, and CLC3^high^ cells treated with Rapa. **P<0.001. vs. Control. ^##^P<0.001. vs. BafA1/Rapa. (E)Co-immunoprecipitation showing the interaction between CLC3 and ATP6V1A (V-ATP subunit). (F) Sphere formation assay of CVC cells with different CLC3 expression levels following a2v-mAb treatment. *P<0.05, **P<0.001. vs. Control. #P<0.05, ##P<0.001. vs. a2v-mAb.
