# Supplementary figures and images for: CLC3 regulates V-ATPase to enhance lysosomal degradation and cisplatin resistance in cervical cancer cells
Source: Cell Death Discov. 2025 Dec 3;12:5. doi: 10.1038/s41420-025-02876-0 (PMC12783824; doi:10.1038/s41420-025-02876-0)

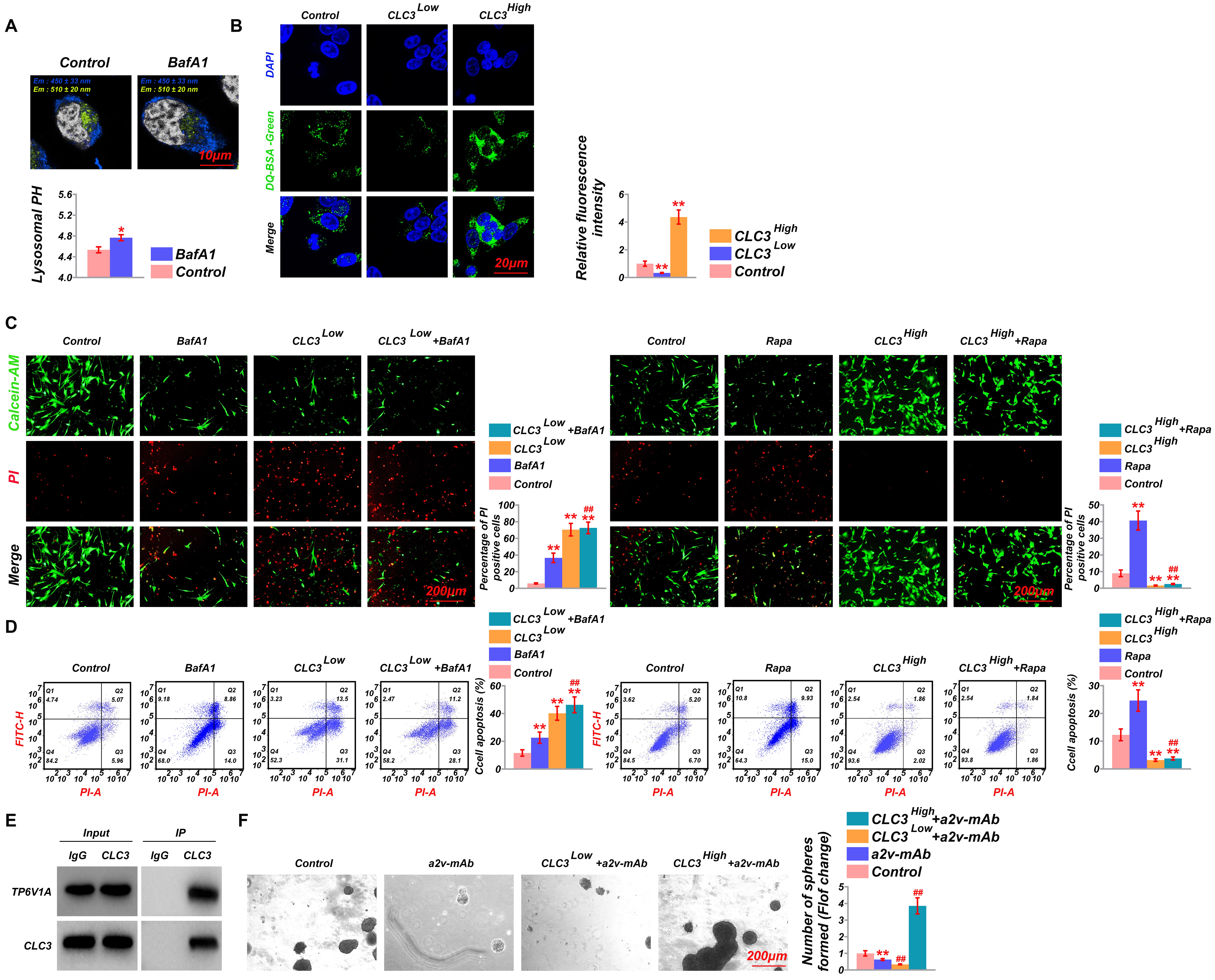

Supplement: Supplementary file 2 — Figure S1 [file 41420_2025_2876_MOESM2_ESM.jpg]
