# Supplementary material for: CLC3 regulates V-ATPase to enhance lysosomal degradation and cisplatin resistance in cervical cancer cells
Source: Cell Death Discov. 2025 Dec 3;12:5. doi: 10.1038/s41420-025-02876-0 (PMC12783824; doi:10.1038/s41420-025-02876-0)
Supplement: Supplementary file 3 — Original Data [file 41420_2025_2876_MOESM3_ESM.pdf]

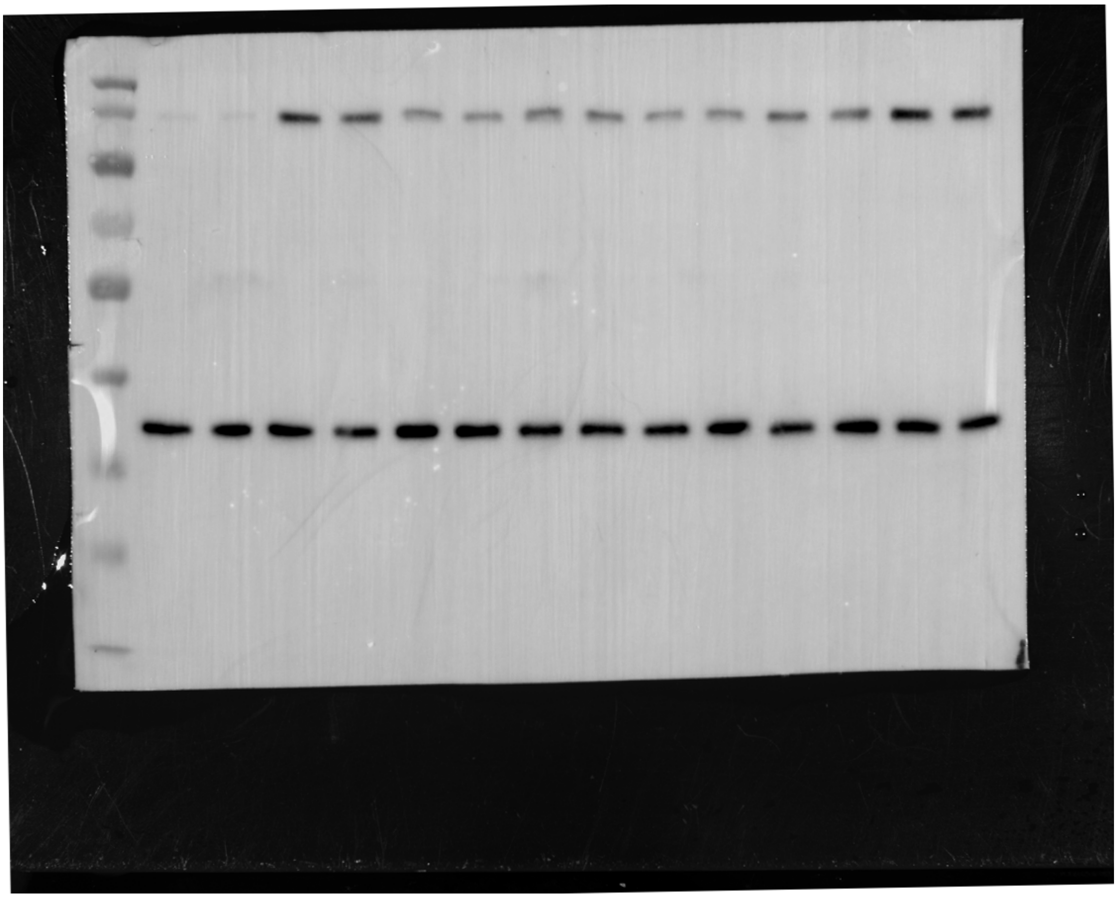

Figure 1B CLC3&GAPDH

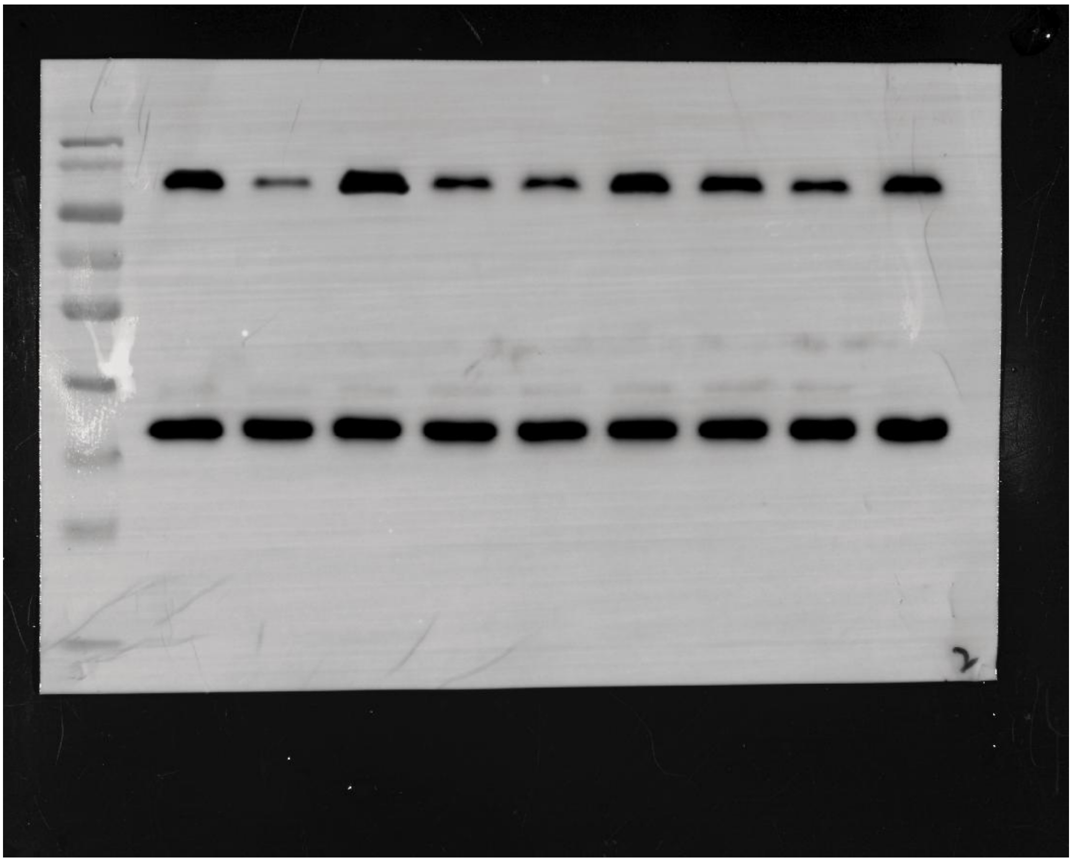

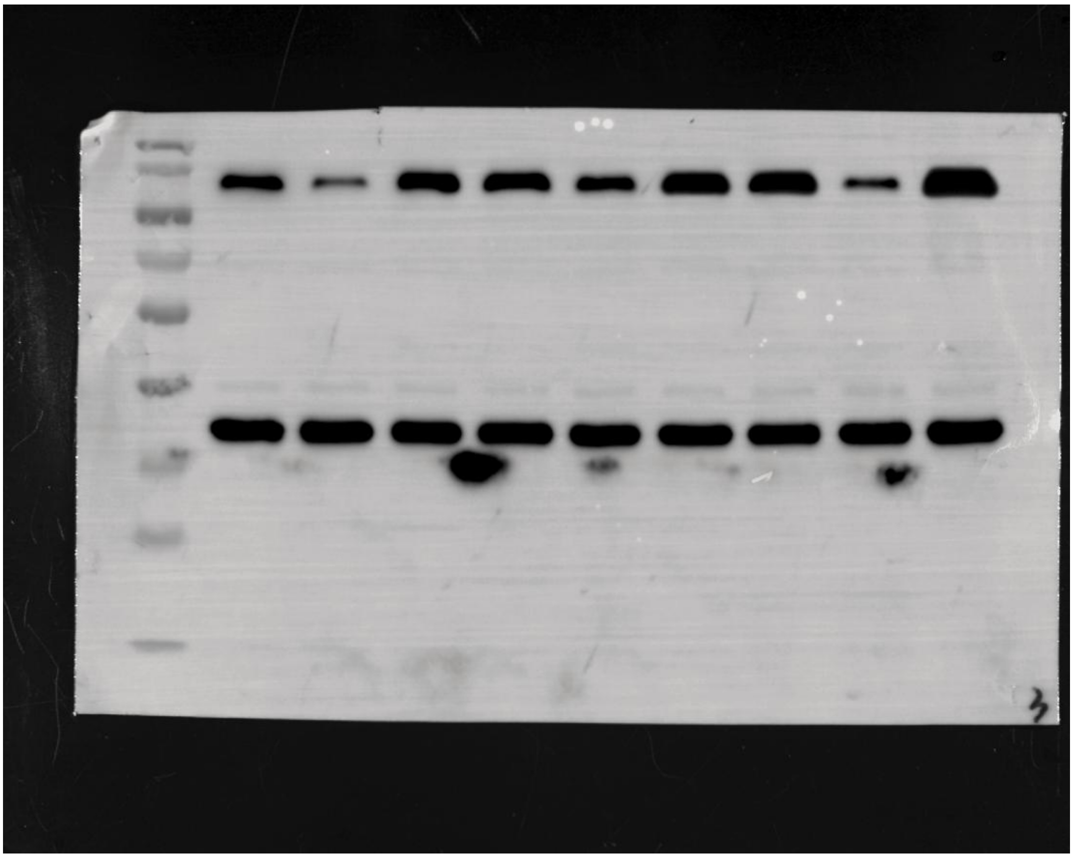

Figure 1D   CLC3&GAPDH

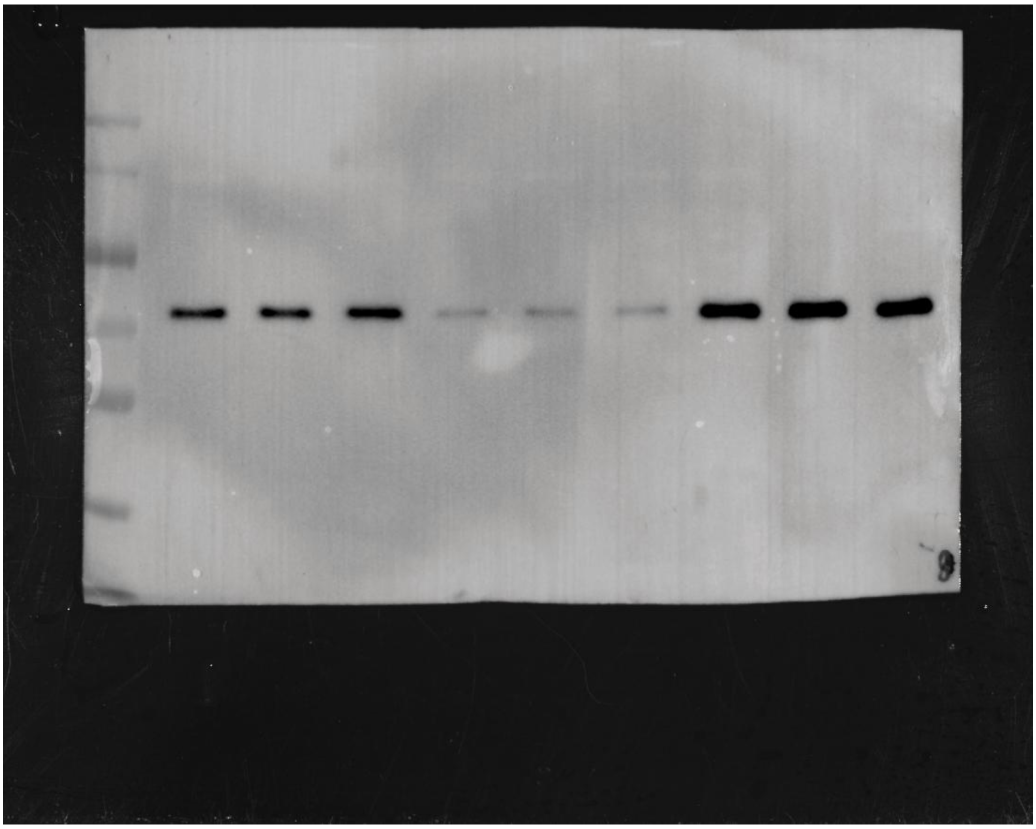

Figure 3A P62

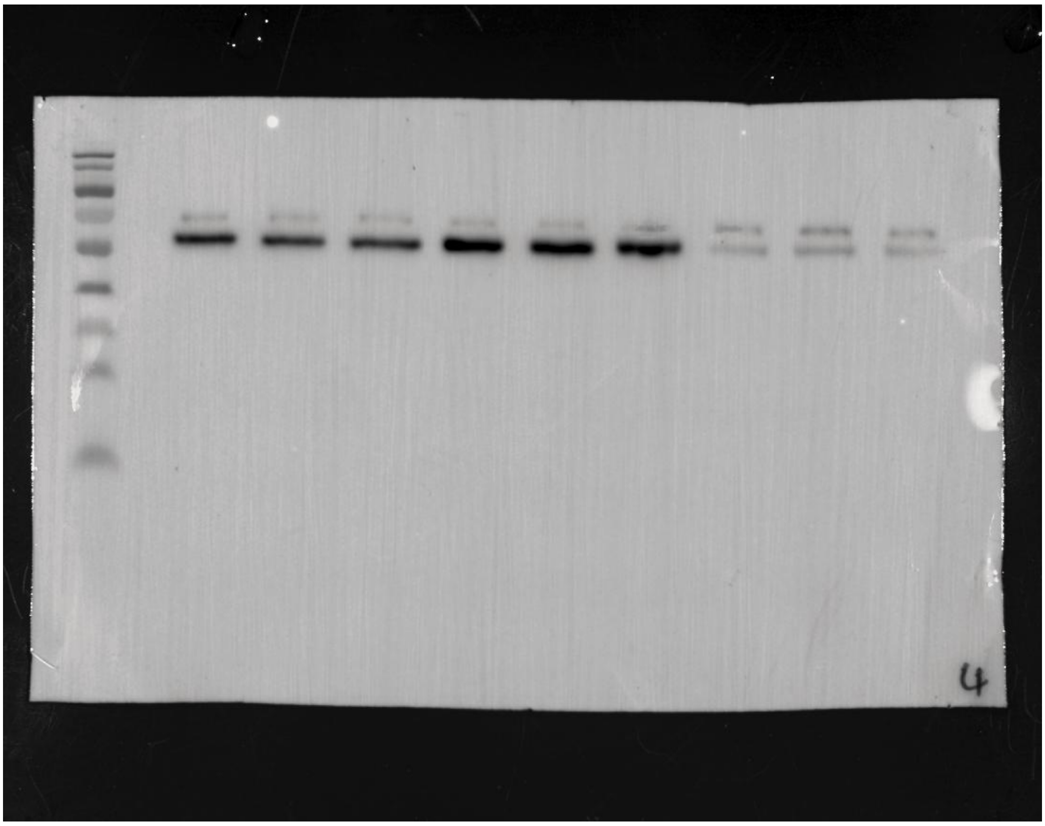

Figure 3A LC3II&LC3I

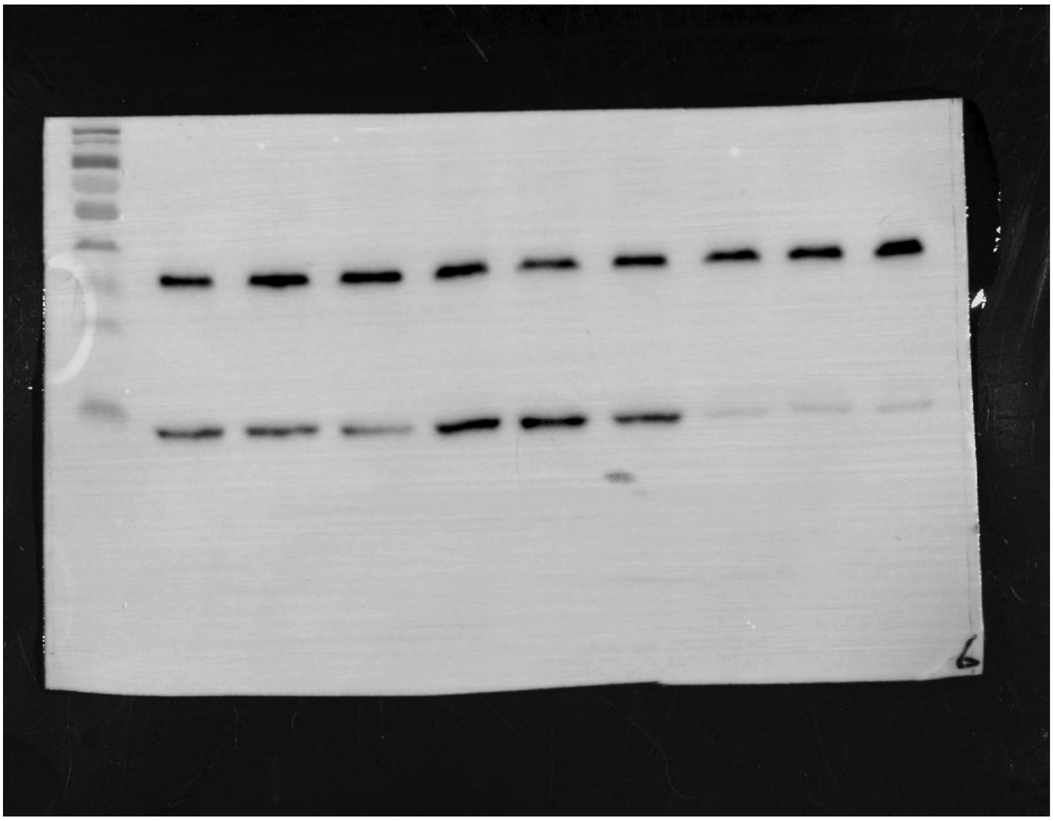

Figure 3A Caspase-3

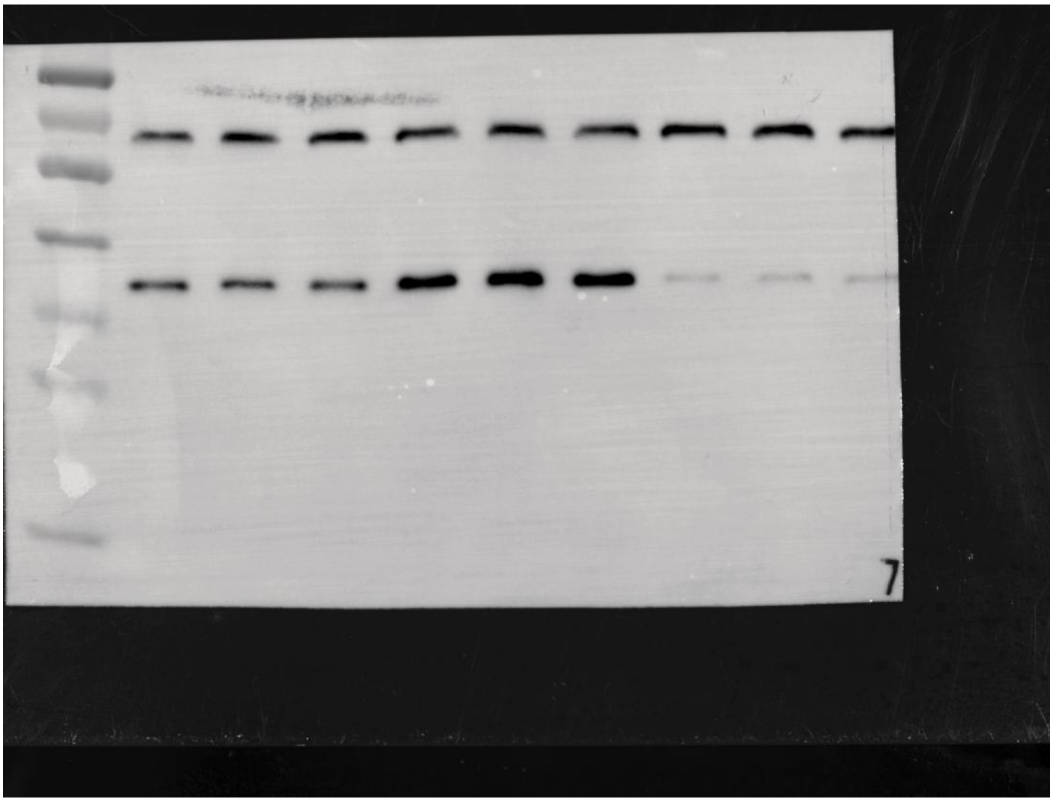

Figure 3A Caspase-8

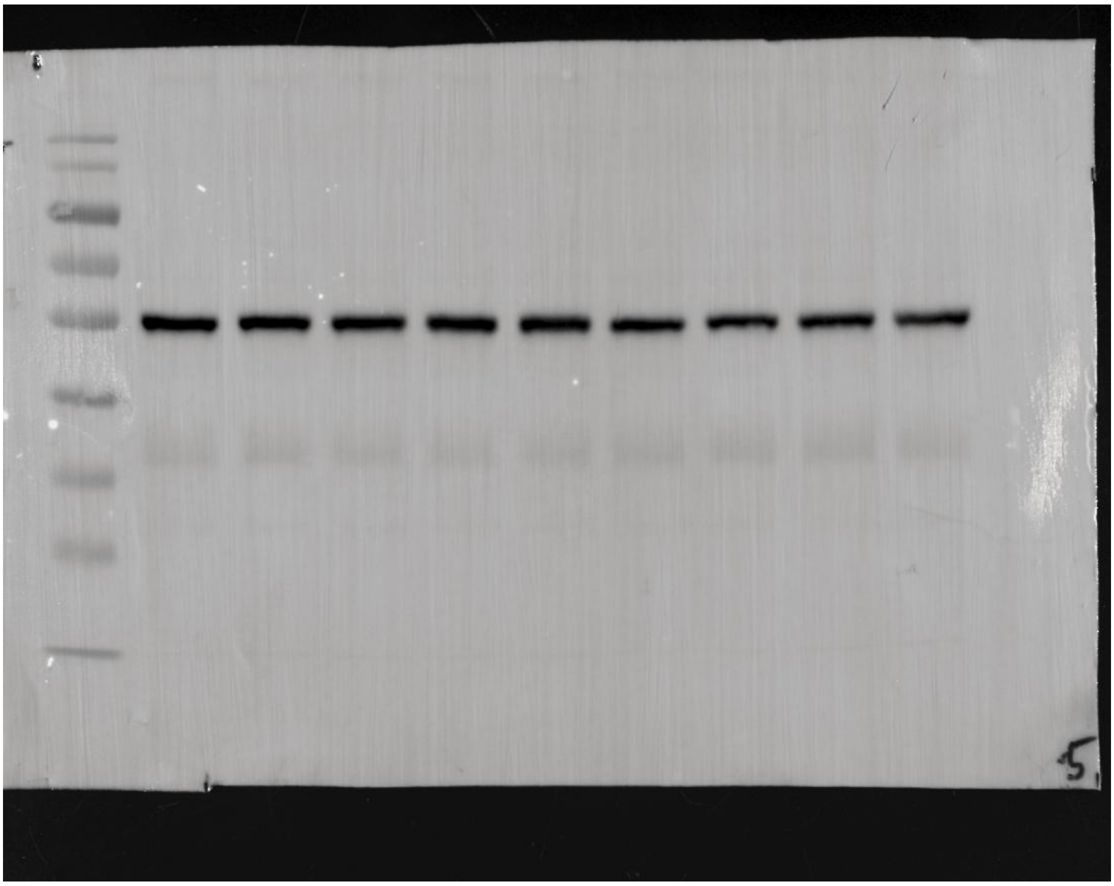

Figure 3A GAPDH
